# Assessments Under Pressure: Interviews With Triage Nurses in Emergency Departments: An Exploratory Descriptive Qualitative Study

**DOI:** 10.1111/jan.70283

**Published:** 2025-10-11

**Authors:** Hugh Gorick, Marie McGee, Toby O. Smith

**Affiliations:** ^1^ University of East Anglia, Norwich Research Park Norwich UK; ^2^ Norfolk and Norwich University Hospitals, Colney Lane Norwich UK; ^3^ University of Warwick Coventry UK

**Keywords:** emergency departments, interviews, nursing, qualitative, triage

## Abstract

**Aims:**

To understand the experiences and decision‐making practices of registered nurses when assessing acuity at triage in emergency departments.

**Design:**

The study utilised a qualitative exploratory‐descriptive design.

**Methods:**

Purposive sampling recruited 11 registered nurses with triage experience from across the United Kingdom. Semi‐structured online interviews, incorporating practice‐based vignettes, were conducted between April and November 2024. Thematic analysis was selected to analyse the data.

**Results:**

Three themes were identified: (1) Pressurised decisions, highlighting the effects of overcrowding, staffing shortages and operational burdens; (2) Holistic assessments, revealing the shift from structured to intuitive decision‐making as nurses gain experience; and (3) Confidence, competence and emotional wellbeing, illustrating the psychological impacts of triage and the importance of experience and support.

**Conclusions:**

This study provides novel insight into how triage nurses navigate acuity assessment in high‐pressure environments. It shows how experience, training and institutional culture influence decision‐making and wellbeing. It identifies key areas for targeted intervention.

**Implications for the Profession and/or Patient Care:**

Triage nurses face substantial cognitive and emotional strain, which may compromise assessment quality and safety. Findings highlight the urgent need for structured triage training, protected time for assessment and access to wellbeing and peer support systems.

**Impact:**

*What problem did the study address?*: A need for current literature exploring the practices and experiences of triage nurses. *What were the main findings?*: Triage nurses experience significant environmental and emotional pressures, develop decision‐making strategies through experience and require enhanced training and support to ensure safe, effective care. *Where and on whom will the research have an impact?*: Findings are relevant to emergency departments internationally, triage nurses, nurse educators and healthcare leaders.

**Reporting Method:**

This study adheared to COREQ reporting guidelines, and a copy of the checklist is attached as Data S1.

**Patient or Public Contribution:**

This study did not include patient or public involvement in its design, conduct, or reporting.

**Summary**

- What does this paper contribute to the wider global clinical community?
  ○ Exploration of triage practices when faced with current emergency department pressures.
  ○ Recommendations to support the safety and development of triage nurses.

## 1 Introduction

Globally, emergency departments (EDs) are facing growing pressures from rising patient demand, constrained resources and workforce shortages (Sartini et al. 2022). Across diverse health systems, emergency nurses report similar challenges, including overcrowding, shortened triage windows and increased clinical and emotional burden (Wolf et al. 2018; Reay et al. 2020). Emergency departments are increasingly characterised by high patient volumes, rising acuity and reduced time for assessment (Wemm and Wulfert 2017; The King's Fund 2024).

Further, these environments can have an impact on the nurses themselves. There is increasing international evidence to suggest that these busy environments are resulting in high levels of stress being experienced by nurses working within emergency care, impacting how they care for patients within these environments (Basu et al. 2020; Wolf et al. 2020; Gorick et al. 2024). Stress has been linked to nurses' abilities to accurately assess patient acuity, resulting in missed cues and reduced quality of assessments (Yuwanich et al. 2015; Wemm and Wulfert 2017). These findings, in conjunction with the increased patient numbers, highlight environments where increased patient volumes and insufficient staffing elevate stress levels among nurses, impairing their ability to perform effective triage and increasing the time taken for nurses to assess, subsequently further increasing wait times and potentially risking patient harm.

## 2 Background

The triage process, as the frontline of emergency care, is particularly vulnerable to these systemic pressures. Assessment of acuity at initial patient presentation is vital to ensuring patients receive treatment in a timely manner, with negative outcomes associated with poor or untimely triage increasing the risk of long‐term harm or mortality (Yancey and O'rourke 2023). International studies have demonstrated that in overcrowded or under‐resourced EDs, nurses often rely on intuition, visual cues and abbreviated assessments to make triage decisions and minimise risk (Bijani et al. 2018; Wolf et al. 2018; Reay et al. 2020). A recent systematic review of international qualitative literature by the authors (Gorick et al. 2023) highlighted how nurses rely on holistic reasoning, clinical intuition and situational awareness to assign acuity, and established a need for further research into how these techniques develop as participants gain experience.

While international research demonstrates that triage nurses face high levels of pressure and often rely on intuition when making decisions, the United Kingdom (UK) presents a distinctive context not qualitatively examined in over 18 years (Edwards 2007). UK emergency departments operate under unique systemic pressures, including the four‐hour performance standard, nationally integrated urgent and emergency care pathways and widespread use of digital triage tools. In addition, the combined impacts of chronic workforce shortages and pressures intensified by the COVID‐19 pandemic have created conditions not necessarily directly comparable to those in other countries (Sartini et al. 2022). Given changes to practice and rising workloads, triage nurses' experiences and decision‐making processes may have evolved, necessitating renewed exploration of their practices. Further, the structural and operational differences between UK and international emergency departments mean that findings from overseas studies cannot be assumed to apply directly to the UK, reinforcing the need for new qualitative research into how triage nurses working within the National Health Service (NHS) experience and enact decision‐making under pressure.

## 3 The Study

This study is part of a sequential series of qualitative studies and follows a wider systematic review of global qualitative literature (Gorick et al. 2023) and a national survey of UK triage nurses (Gorick et al. 2024). The findings of these studies highlighted inconsistencies in training, a growing reliance on clinical intuition and the emotional toll of triage practice. This interview‐based phase explores these findings in greater depth, allowing participants to reflect on how they experience and enact triage when faced with departmental and operational pressures that may impact their abilities to accurately prioritise patients.

### 3.1 Aims and Objectives

The aim of this study is to understand the experiences and decision‐making processes of registered nurses when assessing acuity at triage in UK EDs.

## 4 Methods

The study utilised an exploratory‐descriptive qualitative (EDQ) design rooted in a constructivist paradigm, following guidance from Hunter et al. (2019), with online semi‐structured interviews chosen as the data collection method.

### 4.1 Design

The study is informed by a constructivist epistemology, which holds that knowledge is not objective and fixed, but rather constructed through social processes and individual meaning‐making (Gerrish and Lathlean 2015). EDQ research is aligned to using a constructivist approach due to the focus on the importance of the context of participants and the situation in both ontologies (Doyle et al. 2019). In the EDQ constructivist viewpoint, triage practice is not simply a mechanical application of protocols but is deeply situated within each nurse's unique background, skillset and working environment. However, as noted by Chafe (2017), when performing data analysis in healthcare with EDQ, it is important to keep the results grounded in lower‐level interpretations, both to keep to the aims of the study and maintain its accessibility to those it targets working in the healthcare environments.

This EDQ constructivist lens informed all phases of the study. We used purposive sampling to capture diverse, context‐dependent realities of triage practice. Semi‐structured, participant‐led interviews enabled nurses to construct meaning around their experiences, with vignettes encouraging interpretation shaped by individual context (Lincoln et al. 2017). Inductive thematic analysis prioritised participants' language while recognising the researcher's interpretive role.

### 4.2 Recruitment

This study employed a purposive sampling strategy (Etikan et al. 2016), consistent with exploratory‐descriptive qualitative research and a constructivist orientation, which prioritises contextual depth and diversity of experience over representativeness (Hunter et al. 2019). The interview sample inclusion criteria consisted of registered nurses who currently have face‐to‐face triage as part of their role in UK NHS EDs. Nurses working exclusively in paediatrics, mental health and obstetrics were excluded, as these have different methods of triage.

Sampling sought equal recruitment primarily across two dimensions: geographic area and experience. The aim was to gain representation across the nine sub‐national divisions of England, along with Scotland, Wales and Northern Ireland, to enhance transferability. Informed by findings from Gorick et al. (2024), the study also sought to recruit an equal sample of more and less experienced nurses, defining ‘experienced’ as over three years qualified, reflecting the typical transition from ‘advanced beginner’ to ‘competent’ (Benner 1982), and where nurses are expected to have gained significant clinical experience and completed advanced courses (Royal College of Nursing 2024). This sampling approach did not aim to achieve balance across all subgroups but to ensure sufficient diversity of context and experience to inform a meaningful exploration of how triage is understood and practised within today's NHS and whether that practice evolves with experience.

The target sample size was 9–17 participants. Rather than aiming for data saturation, which assumes thematic redundancy and is often unsuitable for constructivist or variation‐focused designs (Braun and Clarke 2021), the study followed the concept of informational power (Malterud et al. 2015). Informational power considers the adequacy of the sample based on specificity of aim, richness and quality of dialogue, relevance of participants to the research focus and strength of the applied analysis. In this context, a relatively focused research aim, in‐depth participant narratives and alignment between sample and phenomenon of interest meant that a smaller sample could be justified, provided interviews generated nuanced and practice‐rich data.

Participants were recruited through multiple online social media platforms including RCN forums, Twitter and Facebook. Invitations were also emailed to those who indicated they would be interested in being interviewed during the previous survey (Gorick et al. 2024).

Twenty people expressed interest in participating. Six people expressed an interest but did not meet eligibility criteria. Four people expressed interest but were lost to follow‐up, with no reason given. One person expressed an interest but, on further consideration, declined to participate as they felt the study was not suitable for them.

### 4.3 Data Collection

Data collection utilised online one‐to‐one interviews led by the lead researcher (HG) through Microsoft Teams, which were audio recorded and stored for transcription. A topic guide for interviews (Data S2) was developed. The areas of focus were informed by findings of a systematic review (Gorick et al. 2023) and national survey (Gorick et al. 2024). The topic guide started with demographic questions. Following these, the topic guide moved to the main questions, which reflect deeper investigations of findings from previous studies in this series and consisted of: experiences in triage; training for triage; and decision‐making in triage, which was framed around a vignette designed to ground participants' responses in a shared situation. The vignette featured a busy emergency department and presented three patients as they arrived, asking the participants how they would assess them and what actions they would take. The vignette also presented the last patient as arriving during the assessment of the second, to assess responses when conflicting demands occur. The vignette is presented in Data S2.

The topic guide and vignette were validated by assessment from two senior researchers at the University of East Anglia, and through pilot testing with four experienced nurses working at a local ED in a large teaching hospital.

Interviews lasted between 24 and 42 min. Whilst the length of interviews was shorter, the quality of data produced was high, producing rich data transcripts, supporting sampling methods aiming for informational power (Malterud et al. 2015). No repeat interviews were conducted. Interviews were anonymised and transcribed intelligent verbatim by the lead researcher (HG). Sections of the transcription were checked by two other researchers (MMG and TS) to ensure veracity. A complete transcript from their interview was sent to each participant to ensure agreement (Nowell et al. 2017).

### 4.4 Ethics

The study was reviewed and approved by the University of East Anglia Faculty of Medicine and Health Sciences ethics board before commencing, [ETH2324‐1719, granted 26/03/24]. Participants who emailed expressing interest were sent a participant information sheet and consent form, which they were asked to read, sign and return before the interview. To maintain confidentiality, recordings were destroyed after transcription, and all transcripts and quotes were pseudonymised. Individual participant demographics are presented as broadly as possible to support confidentiality. To ensure psychological safety, participants were debriefed after the interviews (Dempsey et al. 2016), with signposting available to local and national services if needed.

### 4.5 Data Analysis

Transcriptions of the interviews were analysed utilising inductive thematic analysis, based on Clarke and Braun (2017). This approach was chosen for its compatibility with both exploratory‐descriptive qualitative methodology and a constructivist paradigm. Transcripts were read and re‐read to ensure familiarity and allow an overview, before being inductively coded by the lead researcher (HG) using NVIVO, which was used not only to store data but also to code transcripts and track analytic decisions. Codes were derived primarily from participants' own language to retain closeness to their meanings, while recognising that interpretation was shaped by the researcher's own clinical and academic background. Field notes taken by the lead researcher (HG) during the interview were used to support the analytic process.

To support credibility and consistency, the accuracy of coding was verified by the research supervisors (MMG and TS), with both coding sections of two transcripts each compared to coding by the lead researcher (HG). While no formal inter‐coder agreement metric was used, the process allowed for dialogue and clarification around meaning and interpretation, with agreement reached through discussion rather than quantification.

**FIGURE 1:**
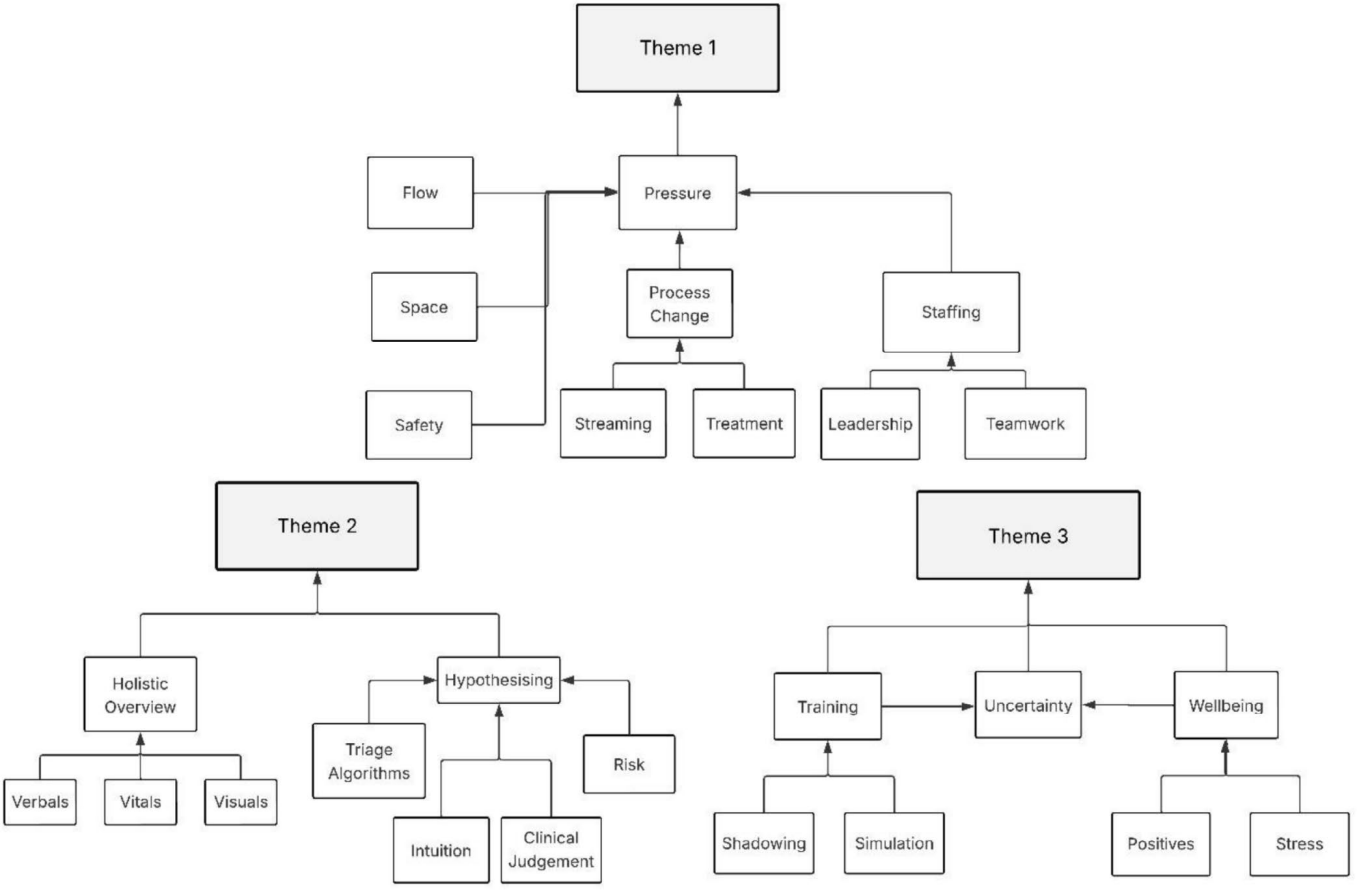
Interview coding tree.

**FIGURE 2:**
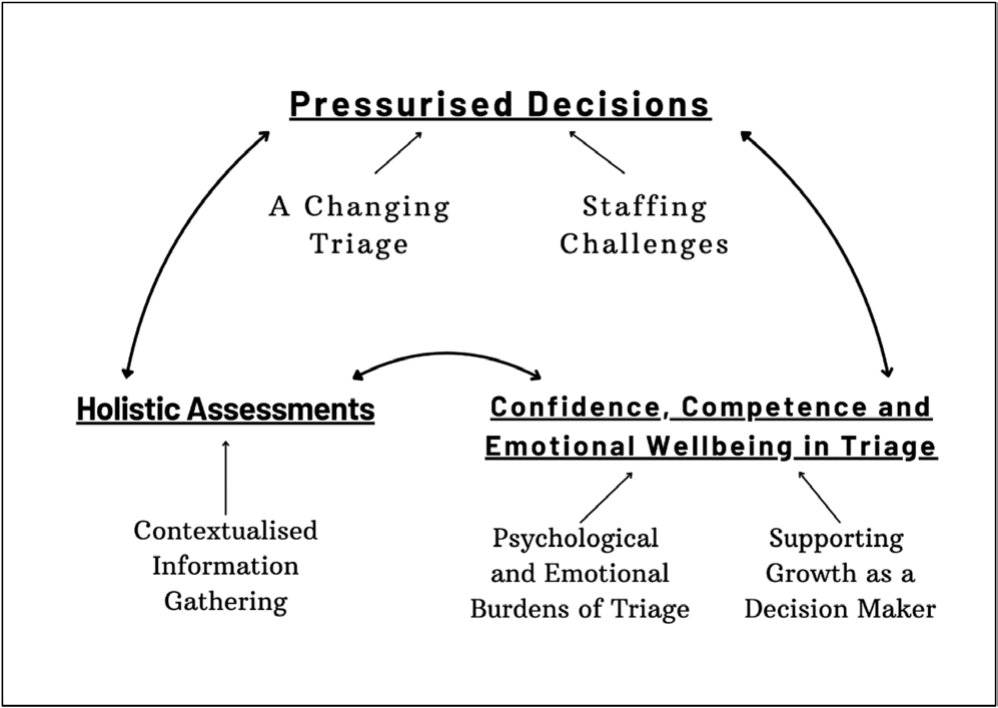
Themes and subthemes.

Themes were developed by grouping related codes and organising them into coherent patterns that reflected both shared and divergent experiences. Themes were reviewed and refined collaboratively through ongoing discussion with the supervisory team (MMG/TS) (Nowell et al. 2017). Final themes and subthemes were confirmed through iterative dialogue between researchers and supported by a coding tree and thematic map (Figures 1 and 2). Participants were offered a copy of the findings if they indicated that they would like to receive one.

### 4.6 Rigour and Reflexivity

Rigour was established through practices consistent with constructivist qualitative inquiry, focused on ensuring trustworthiness rather than positivist criteria such as reliability or validity (Nowell et al. 2017). This included prolonged data engagement, multiple coders, peer debriefing and reflexive practices throughout.

The lead researcher (HG) is a male postgraduate student, holding an MSc and currently undertaking a PhD. He also works clinically in an emergency setting, with prior research experience in triage. This insider status positioned him uniquely within the research: embedded in the professional context under study and responsible for its interpretation. From a constructivist perspective, this dual identity is acknowledged as shaping the research encounter, with knowledge co‐constructed through interaction between researcher and participant (Berger 2015).

While this closeness to the field may introduce interpretive bias, it also enabled rapport with participants, immediate understanding of clinical and organisational context and sensitive follow‐up on implicit cues during interviews. To address potential limitations of this positionality, reflexivity was embedded throughout. HG maintained field notes and a reflexive journal to document assumptions, decisions and emotional responses during data collection and analysis. Regular meetings with supervisors (MMG/TS), who were not practising emergency clinicians, served as peer debriefing, providing alternative perspectives and enhancing critical distance.

Interpretive rigour was supported through coding checks by the supervisory team and discussion of theme development across multiple iterations. Particular attention was given to ensuring participants' meanings were prioritised to avoid any researcher preconceptions. This was supported through inductive coding, collaborative theme development and transparent decision‐making in NVivo, which was used to audit and track analytic decisions, further enhancing confirmability. Two transcripts were independently coded by supervisors and compared with HG's codes to ensure consistency and allow space for divergent interpretations.

All interviews were conducted virtually via Microsoft Teams. While this enabled national recruitment, it required particular attention to rapport‐building, privacy and data quality. Two participants worked in the same hospital as the interviewer, although in a different department. Before interviews, it was made clear that HG was acting in his capacity as a university‐based researcher, not as a clinician or hospital employee, and that all responses would be anonymised and treated in strict confidence. To reduce the risk of response bias, participants were reminded there were no right or wrong answers, and that all insights, whether critical or affirming, were equally valued. During analysis, reflexive notes were used to monitor for any undue influence of prior familiarity, and no discernible difference in tone or openness was observed in these interviews.

## 5 Findings

Eleven triage nurses participated in the interviews between 11th April 2024 and 19th November 2024. Participants were recruited from eight of the 12 geographic regions targeted. An even number of more and less experienced participants was recruited. Demographic characteristics are illustrated in Table 1. Individual demographics are contained in Table 2.

**TABLE 1:** Demographics.

	*N*	%
Gender		
Female	9	82
Male	2	18
Age	30	23–63^b^
Years qualified	4^a^	1–27^b^
Years working in triage	4^a^	1–27^b^
≤ 3 years in triage	5	45
> 3 years in triage	6	55
Highest qualifications		
DipHE	1	9
BSc	7	64
MSc	3	27
Qualifications gained in UK	11	100
Agenda for change NHS band		
5	6	55
6	4	36
7	1	9
Location		
London	2	18
East of England	2	18
Scotland	2	18
West Midlands	1	9
East Midlands	1	9
South West	1	9
Yorkshire	1	9
South East	1	9

^a^ Median.

^b^ Range.

**TABLE 2:** Individual participant demographics.

Participant ID	Location	Gender	Highest qualification	Years working in ED	Years working in triage	Band	Job title
1	East of England	F	BSc UK	4	3	6	Deputy sister
2	South West	F	MSc UK	27	27	7	Advanced nurse practitioner
3	West Midlands	F	DipHE UK	12	11	6	Sister
4	Scotland	F	BSc UK	2	2	5	Staff nurse
5	London	F	BSc UK	4	4	5	Registered nurse
6	East of England	F	MSc UK	2	1	5	Emergency department nurse
7	London	M	BSc UK	1	1	5	Registered nurse
8	East Midlands	F	MSc UK	6	5	6	Charge nurse
9	Yorkshire	F	BSc UK	5	5	6	Deputy sister
10	South East	F	BSc UK	4	4	5	Registered nurse
11	Scotland	M	BSc UK	3	2	5	Staff nurse

Data analysis resulted in the creation of 1045 individual descriptive codes. These were grouped into 26 initial analytic codes. Analysis resulted in the identification of three major themes, two of which had two subthemes and one of which had one. The coding tree is contained in Figure 1, and the final themes and subthemes in Figure 2.

### 5.1 Theme One: Pressurised Decisions

The pressure EDs currently face was a major point of discussion. The first theme considers participants' perspectives of these pressures and how they felt they impact their abilities to assess patients. The subthemes explore how the triage environment has changed toward this pressure, as well as the importance of staffing levels and teamwork.

Participants from across geographic areas highlighted several key environmental stressors that affect their abilities to effectively assess acuity, such as high patient volume, space constraints and the need for rapid decision‐making. High patient numbers were consistently identified as a significant source of pressure, with participants discussing challenges to quickly identify and prioritise patients at risk of deterioration.

You know, you might have a corridor of people sitting there who all need observations and you just have to be like, OK, he's the sickest and prioritise that. So that's stressful. P5 (4 years' experience in triage)

The lack of physical space in departments was frequently cited by participants as a significant barrier to effective triage. While some participants noted their departments had protected triage areas, flow limitations still result in patients returning to crowded waiting areas. These flow limitations were frequently attributed to hospital‐wide flow issues, with participants describing how delays in discharging or admitting patients creates backlogs in the ED. This had a direct impact on triage, both practically and emotionally.

We can't manage to treat these patients, assess these patients and manage these patients because there's because of the lack of space, which can be very frustrating. P9 (5 years' experience in triage)

The waiting room was also identified as a ‘high‐risk’ area by more experienced participants, with overcrowding often leading to compromised patient safety. Participants noted a lack of dedicated staff in these areas added to the stress, highlighting the difficulty of balancing patient safety with managing high volumes.

…but I'm quite alarmed now at our waiting rooms, you know they're overcrowded, they're dehumanised. P2 (27 years' experience in triage)

Overcrowding, limited staffing and spatial constraints were linked to increased clinical risk by participants, often requiring them to balance need for comprehensive assessment against pressure to maintain flow. Participants described how this leads to shortened assessments, seen as a necessary adaptation, though several participants also reflected on the risk of missing cues in these situations, suggesting they lead to inaccurate decisions.

#### 5.1.1 A Changing Triage

The first subtheme highlights how challenges in delivering care within triage have evolved over time in response to increasing pressures. Experienced participants from all geographic areas expressed concerns that triage is now busier with higher proportions of acutely unwell patients, increasing workloads and reducing time available for thorough assessments.

Participants reported significant changes in the composition and acuity of ‘walk‐in’ patients. Historically, the most critically ill patients arrived via emergency services, but this pattern has shifted, with more critically ill patients now self‐presenting. This shift required participants to quickly adapt to the evolving patient profile and be ready for acutely unwell individuals arriving by any means.

…notoriously, the ill‐est patient needs to come in via either helicopter or ambulance, and now they are self‐presenting. P2 (27 years' experience in triage)

This evolving patient profile, alongside rising demand, was seen to place growing pressure on the triage process. An expansion of the nurse's role from acuity assessment to rapid streaming into predefined care pathways was reported by participants. While some viewed this as a necessary adaptation, other participants, particularly those in departments where streaming occurred before triage, expressed concern that decisions based solely on presenting complaint risk missing the broader clinical picture. These participants emphasised that acuity is not always apparent at first glance, and a holistic triage assessment remains essential for safe prioritisation.

Many participants also expressed concerns about how responsibilities have expanded beyond traditional triage tasks, with discussion about how they now involve managing space and patient flow in the department. They highlighted how operational burden often detracts from their primary role of assessing patients, creating significantly increased workloads and reducing their abilities to accurately assess patients.

You spend a lot of time moving people around that would be best put in actually assessing patients at times. P4 (2 years' experience in triage)

Additionally, participants drew attention to how roles have expanded to include more extensive treatment responsibilities, such as administering infusions or personal care. Participants attributed this change to systemic issues like bed shortages, staffing problems and prolonged waits. Yet while these actions were often described as necessary to maintain patient safety and dignity, they also further reduced nurses' capacity for timely and comprehensive assessments.

#### 5.1.2 Staffing Challenges

The second subtheme highlights how staffing levels and perceptions of team support were critical to the abilities to manage triage effectively. How inadequate staffing intensified existing pressures was described by participants across geographic areas and levels of experience, limiting their capacity to assess patients thoroughly and safely. This lack of staff means that triage nurses often had to take on additional roles, looking after patients in waiting rooms and corridors. These responsibilities, while seen as necessary, also contributed to feelings of being overburdened and undervalued.

Even when staffing levels met recommended ratios, many participants feel these numbers are inadequate. The sense of strain was often linked not only to nurse‐to‐patient ratios but also to the broader mismatch between official staffing models and the complex realities of triage work. Frustrations were expressed by participants that because they accommodate the increased workloads, managers feel that short staffing is acceptable despite the pressure it puts nurses under.

I don't think it shows on… the highest hospital level how serious some of these patients who come in are when we're short staffed. Just because we are handling it doesn't mean that we should. P6 (1 years' experience in triage)

Despite these challenges, strong peer relationships and team cohesion were identified by participants as supportive of resilience. The emotional and practical value of working within a supportive team culture was strongly emphasised. Effective communication and collaboration were identified as key components of successful teamwork, allowing sharing of the burden of decision‐making and reducing individual stress. Teamwork also played a crucial role in emotional sustainability, particularly for newer staff adjusting to the demands of the role. Feeling part of a cohesive team helped reduce feelings of isolation and supported in dealing with triage pressures.

…the teamwork really makes a difference. Like, makes you want to keep working there essentially because it has been very stressful and it's a lifestyle. P5 (4 years' experience in triage)

However, these positive team dynamics were difficult to sustain during persistent under‐staffing. Participants noted that as workloads increased and capacity to support one another diminishes, the protective effect of teamwork was perceived to weaken, with implications not only for triage decision‐making, but also emotional capacity and long‐term retention.

### 5.2 Theme Two: Holistic Assessments

The second theme focuses on how triage nurses assign acuity scores, exploring their assessment processes and how their decision‐making evolves with experience. The subtheme examines how nurses structure their assessments.

Less experienced participants typically described using step‐by‐step frameworks, often beginning with general observations before progressing to structured tools such as observation scoring systems or triage algorithms. These methods were seen to provide a clear guide and offer reassurance in the early stages of developing clinical confidence. For some, the algorithmic structure created a sense of safety and objectivity in what was otherwise an uncertain process.

Conversely, more experienced participants described a gradual shift toward more holistic and flexible assessments, incorporating clinical reasoning, visual cues, interpersonal interaction and intuition. This transition reflects an adaptive, experience‐based approach, where nurses moved from procedural knowledge to pattern recognition and synthesis.

I get the course teaches you to put patients into categories and boxes, but course does not make you a triage competent… it's that gut feeling. P3 (11 years' experience in triage)

Perceptions that the structured systems can miss subtle indicators were described by these experienced participants, such as changes in skin colour, behaviour, or non‐verbal cues, that they learn to recognise over time. These were identified as essential to accurate triage, particularly when conventional assessment tools produced ambiguous results. Rather than discarding structure, these participants appeared to layer multiple forms of knowledge, combining formal tools with professional judgement and contextual awareness.

Intuition played a significant role in assessments, especially in cases where multiple methods of assessment gave ambiguous results. Experienced participants relied on intuition as an early warning system, particularly in cases where vital signs seemed stable but something in the patient's appearance raised concerns. However, they also recognised limitations of intuition, acknowledging that appearances can be misleading. Intuition was used as a starting point, followed by further testing or observation to validate and refine clinical impressions.

Risk management was also a key part of their holistic assessment. Rather than only focusing on immediate symptoms, participants described assessing for future deterioration and system‐level risk, such as the likelihood of delayed care or patient deterioration in the waiting room. This influenced their acuity scoring, with the aim of reducing adverse outcomes.

Even though he's stable now chest pain can change, so I'd want to monitor him closely. P11 (2 years' experience in triage)

The combinations of these risk management frameworks with the information from multiple methods of assessment to form an acuity score represented experienced nurses' needs to go beyond structured assessment tools to gain a more comprehensive holistic overview of the patient.

#### 5.2.1 Contextualised Information Gathering

The single subtheme for this theme explores how triage nurses combine different assessment methods to inform acuity decisions, and how their use of these methods becomes more interpretive with experience. While using multiple assessment methods was described by all participants, the way these were applied varies, reflecting different levels of clinical reasoning and contextual sensitivity.

Vital signs were the most commonly discussed method, offering immediate, quantifiable data about a patient's acuity. However, these observations were often taken at face value by less experienced participants, entered into scoring systems without consideration for the underlying pathologies and contexts that may give meaning to the scores. In contrast, these signs were interpreted within a broader clinical and personal context by more experienced participants, treating the numbers not as endpoints but as clues to be understood in relation to the patient's baseline, history or presentation.

Actually you might not be worried about somebody who's got slightly low blood pressure because…you speak to them and they say, oh, that's quite normal for me. Perhaps they're very fit and healthy or they have ongoing dialysis and that's kind of how their body is functioning. P5 (4 years' experience in triage)

This use of depth of examination for context was also evident in discussion of verbal stories. These verbal explorations allowed nurses to gain insight into symptoms, pain levels and other contextual factors that might influence acuity. These histories were more likely to be treated as discrete data points by less experienced participants. More experienced participants described using verbal questioning as a tool for triangulation, exploring inconsistencies, clarifying symptom progression or contextualising risk.

I'd consider different things, the middle‐aged man might have a history of heart disease or risk factors related to that. With the young guy, I'd focus more on his hydration, activity level, or any previous fainting episodes. P8 (5 years' experience in triage)

Participants, both less and more experienced, expressed suspicion toward self‐reported symptoms, particularly in cases like chest pain and substance misuse. They were especially cautious when patients reported high pain levels that seemed inconsistent with their behaviour. Conversely, they also noted instances where patients downplay symptoms, such as with asthma or chest pain. In these cases, visual confirmation often took precedence, and some participants adjusted acuity based on observed signs rather than reported pain levels.

Visual assessments served as an important initial cue for all participants; however, those with less experience described these as brief‐look tests, used to flag obvious concerns such as altered breathing or mobility. With experience, these visual cues became part of a more nuanced, interpretive practice, used not only to validate other findings but also to trigger intuitive concern in cases where other methods offer limited clarity. These quick‐looks evolved into pattern recognition skills, helping experienced nurses detect early signs of serious illness not yet evident in vitals or patient history.

### 5.3 Theme Three: Confidence, Competence and Emotional Wellbeing in Triage

This theme explores how triage nurses experience and construct confidence in their decision‐making, and how this interacts with emotional wellbeing in high‐pressure environments. The subthemes focus on how triage knowledge and confidence develop through training and experience, and how the emotional impact of triage can affect confidence and decision‐making.

Self‐perceived competence played a central role in shaping both triage accuracy and psychological resilience, especially among less experienced participants. They often expressed doubts about their abilities and perceived competence, contrasting with more experienced staff. The importance of confidence in triage decisions was particularly emphasised.

[Lacking confidence] …Can affect the decision that you make when it comes to triage, because it has that knock on effect …And then make my decision, I then doubt myself. P10 (4 years' experience in triage)

A strong link between emotional state and clinical judgement was identified by participants. Feelings of uncertainty often led to risk‐averse behaviours, such as over‐testing or escalating acuity unnecessarily, not out of clinical necessity, but to reduce anxiety. While these decisions were made with patient safety in mind, they reflect concerns about personal responsibility and fear of making incorrect decisions without adequate support.

Confidence was vital in the assessment of acuity but also in managing uncertainty around limited physical space in the triage area. Less experienced participants expressed doubts about prioritising which patients should occupy available spaces, particularly in urgent or ambiguous cases. This hesitation reinforces the emotional burden of making pressured decisions, especially without consistent support or feedback. Various strategies to manage uncertainty were described by participants, including internal rationalisation, where participants mentally review the patient's presentation and potential outcomes to justify their decisions. This helped them logically consider assessments and needs. Collaboration with colleagues was also seen as a key strategy to reduce uncertainty, with all participants recognising its importance.

I know that there's enough medical, like, expertise on a hand to, you know, to come and help assess the patient. P7 (1 years' experience in triage)

While strategies like rationalisation and collaboration were helpful, experience was the most crucial factor expressed by participants in reducing uncertainty and building confidence. All participants reflected on how repeated exposure to triage gradually enhanced their confidence and abilities to manage uncertainty. Over time, they developed an internal sense of calibration that helps them know when to trust their judgement or to escalate concerns, and helped them to stay composed in unpredictable conditions.

#### 5.3.1 Supporting Growth as a Decision‐Maker

Methods of supporting growth as a triage decision‐maker emerged as a strong subtheme. Both training and experience were considered key to developing competence and confidence in triage decision‐making, with participants emphasising the importance of the availability of these supports.

However, significant geographical variation was revealed regarding formal triage training. Some participants had undergone recognised triage courses, others had in‐house training or no training at all, prompting frustration over the lack of structured opportunities. Training was commonly expected to provide clinical insight and deeper assessment skills by participants, but many felt the content available focused on the mechanics of triage algorithms over real‐world triage complexities.

…so I went into that triage day thinking they were going to tell me all the nursey stuff, the red herrings, the red flags to look for all these kind of things. Nothing. They just taught you how to use the triage system on the computer. P6 (1 years' experience in triage)

How triage training has changed was reflected on by experienced participants. They described earlier training as structured and comprehensive, with practical sign‐off before nurses were considered trained. In contrast, current approaches were seen as brief and aimed at getting staff working independently as quickly as possible.

Beyond formal training, wider clinical education was seen to support triage development. Courses in patient assessment, deterioration recognition and life support were seen as indirectly improving triage decision‐making, especially where direct triage training is limited. These broadened clinical thinking and boosted confidence in managing complex cases.

The importance of learning from experience was a key focus for all participants from across the geographic spectrum, though more experienced individuals emphasised the need for it to be combined with formal training. Experience was recognised as essential for building competence, confidence and emotional resilience in triage decision‐making.

It's mostly all been based on my own clinical experiences, practises and learning from other people's experiences too. P1 (3 years' experience in triage)

Experience gained through structured training was highlighted as particularly helpful. Informal shadowing of experienced colleagues is valued early on, providing insight into effective decision‐making. Simulation training was also praised for offering realistic, low‐risk learning. Several participants wanted more simulation opportunities, noting that structured experiential learning had the most lasting impact.

#### 5.3.2 Psychological and Emotional Burdens of Triage

The combination of high workloads and lack of confidence was emphasised by participants as significantly impacting their psychological and emotional well‐being, forming the final subtheme.

Triage was viewed as a high‐pressure environment by participants of all experience levels, from across the UK. This sense of being overwhelmed was often linked to the responsibility of decision‐making under pressure. Participants expressed anxiety about the weight of their decisions and the potential consequences for both patients and themselves. For some, mostly but not exclusively less experienced, this led to a persistent fear of making mistakes, adding to their emotional burden. This was compounded by broader pressures within EDs. The ongoing strain from high patient volumes, inadequate resources and competing priorities often had significant emotional impacts.

…it was just hitting a brick wall, like it was just relentless… And I went home quite like, defeated. Like you just feel like you haven't done the best that you want to do. P10 (4 years' experience in triage)

These conditions affected more than just stress levels. High workload and lack of rest was linked by participants to diminished cognitive capacity and reduced decision‐making ability. The expectation to assess patients continuously, often without protected breaks, was seen as physically exhausting and a threat to clinical safety. Participants reflected that as shifts progressed without rest, they were more likely to question their decisions or miss critical cues.

Several participants across the UK expressed frustration at the disconnect between organisational messaging about wellbeing and their lived experience. While self‐care was emphasised rhetorically, few described meaningful or enforceable support mechanisms. Participants called for greater mental health support, including regular access to counsellors or scheduled check‐ins to help staff manage the emotional challenges of their work.

It's tough to make decisions when you're worried about the consequences, and I think it's important to have some kind of support system in place to help manage that stress. P11 (2 years' experience in triage)

Beyond access to professional mental health services, participants advocated for fostering a workplace culture that encourages openness, and mutual support. Feeling heard and valued was seen as essential to maintaining morale and mental health in this challenging environment.

Despite these challenges, most participants acknowledged rewarding aspects of triage, particularly the intellectual engagement and autonomy of patient assessment. The ability to make independent clinical decisions and contribute meaningfully to patient care provides fulfilment that helped them continue in the role.

## 6 Discussion

This study provides a context‐rich exploration of how triage nurses in UK EDs experience and navigate decision‐making in high‐pressure environments. While earlier international studies have documented challenges in triage, this is the first UK‐based qualitative interview study in over 18 years to focus on the experiences and decision‐making processes of UK triage nurses. The findings offer new insight into the interplay between environmental constraints, clinical reasoning and emotional resilience in shaping triage work.

### 6.1 Pressurised Decision‐Making in Triage

This theme explores how systemic pressures actively shape the clinical reasoning of triage nurses. While the challenges of overcrowding, limited space and staffing shortages in EDs are well documented (The King's Fund 2024; DHSC 2025), our findings extend this understanding by illustrating how these constraints are negotiated in real time and embedded within the cognitive and emotional processes of triage decision‐making. Participants also voiced frustration over the lack of physical space, creating patient flow issues and delays in patient assessment, issues mirrored in broader ED literature (Roscoe et al. 2016; Wolf et al. 2018).

Participants described how pressure demanded constant adaptation with shorter assessments that factored in departmental capacity as much as patient presentation. Similar trends have been noted internationally, with Wolf et al. (2018) observing United States of America nurses using ‘quick‐look’ triage and prioritising departmental flow over individual acuity. Further, Reay et al. (2020) found assessments by Canadian triage nurses were reduced during high demand, pushing clinical boundaries to protect patient safety. These strategies, though often necessary, were seen to increase the risk of error, and this is reflected in the wider literature (Yancey and O'rourke 2023). As a result, triage was experienced not as a neutral act of categorisation, but as a risk‐balancing exercise shaped by context. Nurses routinely weighed the consequences of incomplete information against the pressure to maintain flow, revealing a form of situational reasoning in which decisions were relational, formed in response to both patient and system‐level variables.

A novel contribution of this study is the detailed depiction of triage nurses' expanding operational responsibilities, reflecting wider NHS issues such as bed shortages and under‐staffing (The King's Fund 2024). Participants in this study reported performing non‐triage tasks, including monitoring waiting rooms, moving patients and providing extended care. Operational burdens have been shown to reduce clinical capacity (Michel et al. 2017; Bakhoum et al. 2021), and while these responsibilities were seen as vital to patient safety, they also contributed to moral strain. Nurses described feeling accountable for outcomes shaped by wider system failures, such as prolonged waits or deterioration in the waiting room, leading to emotional fatigue and ethical tension.

These findings challenge conventional definitions of triage as a discrete clinical task. Instead, triage emerges as a dynamic, systems‐facing role requiring continuous negotiation between competing risks. Where protected triage spaces and adequate staffing were available, participants reported more focused assessments and reduced stress, highlighting the role of environmental design in supporting clinical reasoning. We recommend that EDs recognise and resource the full scope of triage work. This includes protected triage areas, staffing models that reflect operational burden and practical support for managing flow. These structural changes are aimed at safeguarding decision quality and reducing the psychological toll of high‐stakes, high‐pressure triage practice.

### 6.2 Holistic Assessments and the Role of Experience

The second theme, holistic assessments, explores how triage nurses develop their decision‐making skills over time. Less experienced nurses tended to rely on structured assessment tools, while more experienced nurses adopted a more clinical‐reasoning‐based holistic approach. The ability to synthesise and interpret complex information is a hallmark of expertise, as originally described by Benner (1982) and further explored in more recent studies of emergency triage nurses (Bijani et al. 2018; Reay et al. 2020). This study builds on those findings, showing how experienced nurses integrate multiple data points to form working diagnoses and assign acuity scores more quickly and confidently. The shift from structured to intuitive decision‐making is well documented (Benner 1982; Levis‐Elmelech et al. 2022; Gorick et al. 2023). This study builds on those insights by contrasting how less and more experienced UK nurses integrate data, context and uncertainty into decision‐making.

The findings of this study highlight how less experienced nurses focused heavily on structured tools, reflecting their unfamiliarity with triage and a reliance on guidelines to compensate for limited experience. Their assessments followed methodical, stepwise processes, which agree with previous studies noting their uncertainty when working outside of triage protocols (Bijani et al. 2018; Reay et al. 2020). In this study, structured tools were seen as a vital safety net for those still developing intuition and clinical reasoning. However, this reliance also constrained their capacity to respond flexibly to ambiguous or atypical presentations. The algorithmic frameworks provided consistency but limited opportunities for adaptation, often reducing complex clinical encounters to a checklist of symptoms. Rather than engaging with the broader interpretive demands of triage such as synthesising conflicting cues or assessing risk in context, less experienced nurses tended to defer to the perceived objectivity of scoring systems. This suggests that at early stages of triage development, cognitive load and fear of error may lead to a narrowing of clinical focus, where the primary goal becomes rule adherence rather than risk evaluation.

In contrast, experienced nurses preferred intuitive decision‐making, feeling that algorithms were insufficient. They emphasised drawing on a broad range of cues: visual, verbal, historical and vital signs, to build a holistic picture, supporting earlier findings (Roscoe et al. 2016; Bijani et al. 2018). They asked more targeted questions to contextualise symptoms and interpret findings, reflecting deeper clinical reasoning. This reflects a shift from rule‐based cognition to pattern recognition and hypothesis‐driven assessment, where data is actively interpreted. Intuitive judgement emerged not as guesswork, but as reasoning grounded in experience and situational awareness. This qualitative shift in processing warrants further study, particularly regarding the roles of confidence, experience and environment.

### 6.3 Confidence, Competence and Emotional Well‐Being

The third theme, Confidence, Competence and Emotional Well‐being, highlights the psychological and emotional burdens associated with triage. This study found that emotional strain arises from both external pressures, such as overcrowding and resource limitations and internal pressures, including the expectation to make swift, accurate decisions. This is consistent with studies identifying decision‐making under pressure as a significant stressor for ED nurses (Wolf et al. 2018; Reay et al. 2020). Self‐doubt and anxiety, combined with the rapid pace of triage, can compromise accuracy and timeliness, potentially affecting patient safety (Yancey and O'rourke 2023). In this study, emotional uncertainty emerged not as a personal flaw, but as an adaptive response to working without sufficient support.

Participants also described the long‐term emotional toll of triage, including burnout and feelings of defeat. These findings mirror broader research on trauma and stress among emergency staff (Basu et al. 2020; Wolf et al. 2020). Lack of breaks and limited self‐care was highlighted as key contributors to cognitive fatigue and increased error risk (Yuwanich et al. 2015). Many participants in the present study reported feeling drained by the demands of their work, with some highlighting the need for more structured support. Emotional exhaustion was seen not just as a result of workload, but as a systemic consequence of environments that prioritise urgency over recovery. The gradual erosion of resilience affects both wellbeing and clinical judgement, reinforcing the need for organisations to prioritise psychological safety as part of safe practice.

The relationship between confidence in decision‐making and the availability of advanced triage training highlights a significant tension in current educational approaches. Rather than developing the full spectrum of triage competencies, existing training was seen by participants to prioritise algorithmic knowledge at the expense of intuitive and experience‐based judgement. Previous work (Recznik 2018; Khorram‐Manesh 2025) supports the importance of robust training in building confidence and competence. The present and prior studies (Gorick et al. 2023, 2024) show that nurses value clinical judgement and intuition, suggesting training should reflect triage realities by fostering both holistic assessment skills and confidence.

Experience was a critical factor in building confidence. Over time, participants developed internal calibration, the ability to assess acuity, tolerate ambiguity and know when to trust instinct or escalate. This mirrors Benner's (1982) model of expertise and was often supported by informal learning through discussion, observation and cumulative exposure. However, the development of confidence was seen as slow and uneven, shaped by access to feedback, team culture and the level of operational pressure. Bridging formal training with experiential learning could help ensure triage decisions are both safe and confident. Shadowing and simulation were identified by participants as particularly useful methods for gaining both knowledge and experience, an approach supported by prior research (Recznik 2018), and organisations may consider implementing these to help improve their triage processes.

### 6.4 Strengths and Limitations

This study has several notable strengths. To our knowledge, it is the only interview‐based research within the past 18 years to explore the experiences and decision‐making practices of UK triage nurses. A key strength lies in its use of semi‐structured interviews grounded in a constructivist paradigm, enabling participants to articulate how they experience and interpret triage in practice. The exploratory‐descriptive qualitative design, combined with a focus on informational power rather than saturation, generated rich, context‐sensitive insights aligned with the study's aims.

However, limitations exist. The self‐selected sample may introduce bias due to participants' prior interest in the topic, resulting in answers that reflect beliefs as opposed to practices. All care was taken in the discussion to ensure it remained grounded in practice, supported by a practice‐based vignette to centre discussion. Further, the study's findings and recommendations were triangulated against earlier studies in this series and the wider literature to ensure congruence.

The lead researcher's insider status as a clinician was another potential source of interpretive bias. This was addressed through regular reflexive discussions, supervisory input throughout analysis and comparisons between our findings and the wider literature. The constructivist orientation of the study recognises the co‐construction of meaning between researcher and participant and treats this as a feature rather than a flaw of the research process.

The purposive sampling strategy aimed to capture variation across UK regions and levels of experience, aligning with the constructivist assumption that triage practices are context shaped. While a wide range of experience and regions were represented, some UK areas were not included, limiting how far the findings speak to national variation. That said, transferability, not generalisability, was the intended outcome, and detailed contextual information is provided to support interpretation. Further research would strengthen and build upon these findings.

### 6.5 Implications for Policy and Practice

The findings of this study have several implications. Most urgently, participants strongly highlighted the impact that systemic issues such as overcrowding and staffing shortages have on their abilities to work safely. While individual nurses can adapt, urgent systemic solutions are needed to reduce the pressures on EDs, improve patient safety and reduce practitioner burnout, reflecting recent UK NHS plans (DHSC 2025; England 2025). A relatively easy, effective intervention identified was having dedicated and protected triage areas.

Participants in this study also identified operational burdens that impact patient assessments. Less experienced nurses, in particular, expressed uncertainty about managing these added responsibilities. Reducing these burdens where possible and integrating their management into emergency nurse training would better support decision‐making.

Further to this, participants in this study strongly expressed a need for improved training programmes that go beyond the use of algorithmic tools and focus on developing holistic and advanced assessment skills. The participants suggested simulation‐based training and peer mentoring could play a key role in preparing nurses for the complexities of triage work. Some experienced nurses noted that previous triage training pathways had supported their skill development. Structured education that fosters advanced critical thinking, aligned with the UK emergency skills framework (Royal College of Nursing 2024), would benefit.

Finally, and most importantly for staff safety, participants called for greater support for the emotional well‐being of triage nurses. This could include regular access to mental health services, protected break times and a workplace culture that encourages openness and mutual support, in line with recent government recommendations (DHSC 2025).

### 6.6 Future Research

Several areas for further investigation arise from this study. Broader UK‐based and multisite studies to address recruitment limitations identified would benefit. Greater, in‐depth exploration of the experiences and decision‐making processes, with particular focus on transitions as nurses gain experience, would also support the findings of this study. Comparing the influence of different triage methods may also be valuable.

The discussion identified increased operational burdens described by the participants relating to managing space and flow. Further research to investigate the extent, impact and mitigations of these burdens for triage nurses would aid their triage practices. Furthermore, research could investigate the effectiveness of different support strategies, such as peer support programmes or mindfulness training, in reducing the psychological burden of triage work.

The discussion also highlighted a need for structured, holistic triage training, with simulation and mentoring highlighted as promising methods. Research into how such training can be developed and implemented would help identify valuable ways of developing triage nurses' skills to support both their competency and confidence.

## 7 Conclusion

This study has shed light on the complex and demanding nature of triage work in UK EDs. The findings highlight significant pressures perceived by triage nurses, the importance of experience in developing holistic assessment skills and the emotional toll of working in a high‐pressure environment. By addressing these challenges through improved training, systemic changes and greater support for nurse well‐being, it may be possible to enhance both the quality of patient care and the job satisfaction and retention of triage nurses. The interconnectedness of the themes reinforces the need for a holistic approach to addressing the challenges faced by triage nurses, one that considers the clinical, environmental and psychological factors that impact their decision‐making and well‐being.

## Author Contributions

All authors have agreed on the final version and meet at least one of the following criteria: (1) substantial contributions to conception and design, acquisition of data, or analysis and interpretation of data; (2) drafting the article or revising it critically for important intellectual content.

## Conflicts of Interest

The authors declare no conflicts of interest.

## Supporting information


**Data S1:** jan70283‐sup‐0001‐DataS1.pdf.

**Data S2:** jan70283‐sup‐0002‐DataS2.docx.

## Data Availability

The data that support the findings of this study are available on request from the corresponding author. The data are not publicly available due to privacy or ethical restrictions.
