# Supplementary material for: Assessments Under Pressure: Interviews With Triage Nurses in Emergency Departments: An Exploratory Descriptive Qualitative Study
Source: J Adv Nurs. 2025 Oct 11;82(6):6515–28. doi: 10.1111/jan.70283 (PMC13176718; doi:10.1111/jan.70283)
Supplement: Supplementary file 2 — Data S2: jan70283‐sup‐0002‐DataS2.docx. [file JAN-82-6515-s001.docx]

**Supplementary File 2 – Interview Schedule**

1. Introductions – researcher will introduce self and explain the process of the interview.

**Recording starts**

Researcher reads the following:

*Thank you for agreeing to take part in this interview. Before we start can you confirm that you have read the participant information sheet and completed and returned the consent form.*

*I am going to take you through a vignette and ask you questions about your decision-making processes. Please note this is not a test, there is no right answer, and we are interested in your actual and honest reactions to the situation. Try and answer as you would in practice with real patients in front of you. As the vignette comes up, would you like me to read it out loud, or would you prefer to read it off the screen?*

1. Initial Demographic questions

- Gender
- Age
- Highest qualification and where qualifications gained (UK or abroad)
- Years working in ED
- Years working in triage
- Band and job title

1. **First Question** – Tell me about your experiences when triaging patients?

*Follow up questions around this topic may include:*

- Does a triage algorithm aid your assessments?
- How does working in triage make you feel?
- Any specific cases that have stuck in your memory?

1. **Second Question** – How does your training and experience inform your work in triage?

*Follow up questions around this topic may include:*

- Tell me about your training for triage?
- How often do you receive triage training?
- How does your experience influence your assessments?

Read first vignette to participant and display on screen

*It’s a busy night in the emergency department, and you are short on both staff and space. You have one assessment space, and resus and all other trolleys are full.*

*A middle-aged man walks into the emergency department with complaints of chest discomfort. He describes it as a sharp ache that comes and goes but is unable to pinpoint exactly when it started. He denies any shortness of breath, sweating, or radiation of pain.*

1. **Third Question** – Talk me through your decision-making processes about this patient.

*Follow up questions around this topic may include:*

- Can you tell me what is important to you?
- Can you tell me what is least important?
- What is most helpful when making decisions?
- What is least helpful when making decisions?

Read second vignette to participant and display on screen

*As you are finishing with the first patient, an 18 year old boy is brought in by his friends after collapsing during a walk. He was walking ahead when he suddenly fell to the ground, unconscious. Upon arrival, he is alert and oriented but complains of feeling dizzy and lightheaded. His friends report that he had been pushing himself hard during the walk and may not have been adequately hydrated. There are no visible signs of injury*

1. **Fourth Question** – Talk me through your decision-making processes about this patient.

*Follow up questions around this topic may include:*

- Can you tell me what is important to you?
- Can you tell me what is least important?
- What is most helpful when making decisions?
- What is least helpful when making decisions?
- How does this triage compare to the previous patient?

Read third vignette to participant and display on screen

*Halfway through assessing the teenager, an elderly woman is wheeled in on a stretcher, clutching her chest and struggling to breathe. She's accompanied by her husband, who looks on with concern as she gasps for air. She has a history of chronic obstructive pulmonary disease (COPD) and has been experiencing worsening shortness of breath over the past few hours.*

1. **Fifth Question** – Talk me through your decision-making processes about this patient.

*Follow up questions around this topic may include:*

- Can you tell me what is important to you?
- Can you tell me what is least important?
- What is most helpful when making decisions?
- What is least helpful when making decisions?
- How would you manage the lack of space?
- How does this triage compare to the previous two?

1. **Sixth Question** – How do you feel about managing all of these patients together?

*Follow up questions around this topic may include:*

- What could cause potential conflicts?
- How might these be avoided?
- What actions would you take to help the situation?

**Recording Stops (may be started again at request of participant)**

1. Debrief the participant – Discuss the interview, how they felt it went, if they need to discuss how they feel about any sensitive topics, where they can go if they experience any issues. Ask if they have any questions for the researcher.
